# Role of H-Ras/ERK signaling in carbon nanotube-induced neoplastic-like transformation of human mesothelial cells

**DOI:** 10.3389/fphys.2014.00222

**Published:** 2014-06-12

**Authors:** Warangkana Lohcharoenkal, Liying Wang, Todd A. Stueckle, Jino Park, William Tse, Cerasela-Zoica Dinu, Yon Rojanasakul

**Affiliations:** ^1^Department of Pharmaceutical Sciences, West Virginia UniversityMorgantown, WV, USA; ^2^Health Effects Laboratory Division, Pathology and Physiology Research Branch, National Institute for Occupational Safety and HealthMorgantown, WV, USA; ^3^Department of Medicine, Mary Babb Randolph Cancer Center, West Virginia UniversityMorgantown, WV, USA; ^4^Department of Chemical Engineering, West Virginia UniversityMorgantown, WV, USA

**Keywords:** mesothelial cells, H-Ras, SWCNT, ERK, cortactin, cell transformation

## Abstract

Rapid development and deployment of engineered nanomaterials such as carbon nanotubes (CNTs) in various commercial and biomedical applications have raised concerns about their potential adverse health effects, especially their long-term effects which have not been well addressed. We demonstrated here that prolonged exposure of human mesothelial cells to single-walled CNT (SWCNT) induced neoplastic-like transformation as indicated by anchorage-independent cell growth and increased cell invasiveness. Such transformation was associated with an up-regulation of H-Ras and activation of ERK1/2. Downregulation of H-Ras by siRNA or inactivation of ERK by chemical inhibitor effectively inhibited the aggressive phenotype of SWCNT-exposed cells. Integrin alpha V and cortactin, but not epithelial-mesenchymal transition (EMT) transcriptional regulators, were up-regulated in the SWCNT-exposed cells, suggesting their role in the aggressive phenotype. Cortactin expression was shown to be controlled by the H-Ras/ERK signaling. Thus, our results indicate a novel role of H-Ras/ERK signaling and cortactin in the aggressive transformation of human mesothelial cells by SWCNT.

## Introduction

CNTs share some common properties with the carcinogenic asbestos fibers, including high aspect ratio, biopersistence and route of exposure, which raises a major concern about their potential carcinogenicity. A recent study by Sargent et al. indicated the tumor-promoting effect of CNTs (Sargent et al., 2014), while several other studies suggested the potential mesothelioma pathogenicity of this nanomaterial (Takagi et al., 2008, 2012; Sakamoto et al., 2009). At the cellular level, CNTs were shown to induce malignant transformation of human lung cells upon chronic exposure (Wang et al., 2011, 2014; Lohcharoenkal et al., 2013). In a recent study, we reported neoplastic transformation of human pleural mesothelial cells by chronic CNT exposure and demonstrated the role of matrix metalloproteinase (MMP)-2 in the process (Lohcharoenkal et al., 2013). Since our study has indicated the induction of proto-oncogenes by CNTs (Wang et al., 2014) and since *H-Ras* oncogene has been demonstrated to be involved in the DNA damage signaling induced by CNTs (Tong et al., 2011), we asked if H-Ras plays a role in the invasive transformation of CNT-exposed human mesothelial cells.

*Ras* oncogene family has been extensively studied during the past few decades. Ras protein is a major regulator of various pathological and physiological processes that control cell proliferation, differentiation and survival. Ras GTPase family proteins are critical players in many signaling networks, connecting a great variety of upstream signals to an even wider set of downstream effector pathways that control numerous cellular functions including cell cycle progression, growth, migration, cytoskeleton dynamic, apoptosis and senescence (Tong et al., 2011). Ras is a membrane-associated guanine nucleotide-binding protein that is normally activated in response to the binding of extracellular signals such as growth factors, receptor tyrosine kinases, T-cell receptors, and phorbol myristate acetate. It acts as a binary signal switch between ON and OFF states. In the resting state, Ras is tightly bound to guanosine diphosphate (GDP), which is exchanged for guanosine triphosphate (GTP) upon binding of extracellular stimuli to cell membrane receptors. In the GTP-bound form, Ras interacts specifically with effector proteins and initiates downstream cascades. To return to the inactive OFF state, Ras cleaves off the terminal phosphate moiety by the intrinsic GTPase reaction and the remaining GDP-bound Ras is no longer able to interact with effectors (Kolch, 2002). Reactive free radicals and cellular redox stress have also been proposed to directly activate Ras. Nitric oxide promotes the direct post-translational modification of Ras by S-nitrosylation at Cys118. This modification results in stimulation of guanine nucleotide exchange, possibly by destabilization associated with other effectors, leading to transduction of Ras mediated signals (Diaz-Meco et al., 1994). Three members of the Ras family, Harvey-Ras (H-Ras), Kirsten-Ras (K-Ras), and N-Ras, are known to be activated in human tumors (Lowy and Willumsen, 1993; Stites and Ravichandran, 2009). The amino-terminal 85 amino acids are identical and the middle 80 amino acids exhibit an 85% homology between the Ras proteins, whereas the carboxyl-terminal sequence is highly divergent (Barbacid, 1987; Boguski and McCormick, 1993). Up to about 30% of all human tumors carry some forms of alteration in the canonical *Ras* genes. The biological effects of Ras proteins are mediated through the activation of several downstream effectors, including Raf, Rac, phosphatidylinositol 3-kinase (PI3K) and Ral (Marshall, 1996). Ras stimulates serine/threonine kinase Raf, followed by activation of the downstream kinase MAPK/ERK kinase (MEK), which in turn phosphorylates extracellular signal-regulated kinases (ERKs) (Kyriakis et al., 1992). In addition to the Ras/Raf/ERK pathway, the small GTPase Rac and PI3K are involved in the mitogenic and oncogenic effects of Ras (Joneson et al., 1996). PI3K is activated by G-protein-coupled receptors in response to extracellular stimuli or by direct interaction with Ras (Kapeller and Cantley, 1994; Rodriguez-Viciana et al., 1994).

Although these Ras proteins share many common signaling pathways leading to similar cellular responses, studies have clearly demonstrated the unique roles of Ras family members in physiological and pathological conditions. Moon *et al*. reported H-Ras, but not N-Ras, induced invasive and migrative phenotypes by activating p38 and ERK signaling pathways, whereas both induced transformed phenotype in human breast epithelial cells through an up-regulation of MMP-2 (Moon et al., 2000). In laryngeal carcinoma, a weaker tumorigenic effect of N-Ras vs. H-Ras and K-Ras was reported (Kiaris and Soandidos, 1995). In this study, we investigated the role of H-Ras in SWCNT-induced neoplastic transformation of human mesothelial cells and evaluated the downstream targets of H-Ras signaling in the transformed cells.

## Materials and methods

### Cell culture and SWCNT exposure

Human pleural mesothelial MeT-5A (ATCC® CRL9444) cells were acquired from American Type Culture Collection (Manassas, VA) and maintained in M199 medium (Life Technologies, Grand Island, NY) with 5% fetal bovine serum (FBS), 2 mM L-glutamine, 100 U/mL penicillin/streptomycin, 1 μg/mL EGF and 50 μg/mL hydrocortisone. Human peritoneal mesothelial LP-9 cells were obtained from NIA Aging Cell repository (Camden, NJ) at passage 5 and maintained in a medium containing a 1:1 ratio of M199 and Ham's F-12 nutrient mixture (Life Technologies, Grand Island, NY), supplemented with 10% FBS, 10 ng/mL EGF, 0.4 μg/mL hydrocortisone, 100 U/mL penicillin/streptomycin and 2 mM L-glutamine. Cell cultures were performed in a humidified atmosphere of 5% CO_2_ at 37°C. SWCNT, synthesized by using a high-pressure carbon monoxide disproportionate process (HiPCO), were obtained from Carbon Nanotechnology (CNI, Houston, TX). Elemental analysis of the supplied CNT by nitric acid dissolution and inductively coupled plasma-atomic emission spectrometry (ICP-AES, NMAM #7300) showed that SWCNT were 99% elemental carbon and contained less than 1% w/w of contaminants.

Cells were continuously exposed to SWCNT at various surface area concentrations of 0.02, 0.06, and 0.2 μg/cm^2^ for 2 months according to the method previously described (Sargent et al., 2014). Briefly, 0.1 mg/mL stocks of SWCNT in phosphate buffer saline (PBS) containing 1% FBS were sonicated and diluted in media (0.1 μg/mL) prior to cell exposure. Cultured MeT-5A and LP-9 cells were exposed to the dispersed CNT every 3 days following a PBS wash and passaged once per week.

### Soft agar colony formation assay

Soft agar assay was performed as previously described (Ottestad et al., 1988). SWCNT-exposed cells at 3 × 10^4^ cells were mixed with the culture medium containing 0.5% agar to the final concentration of 0.33% agar. Cell suspension was immediately plated onto the dish coated with 0.5% agar in culture medium. Colonies were examined under a light microscope (Leica DM, IL) after 2 and 4 weeks.

### Cell invasion assay

Cell invasion was determined in BD Matrigel® invasion chamber (BD Biosciences, NJ). Briefly, cells at the density of 3 × 10^4^ cells per well were seeded into the upper chamber of the Transwell® unit in serum-free medium. The lower chamber of the unit was added with a normal growth medium containing 5% FBS. The unit was incubated at 37°C in a 5% CO_2_ atmosphere for 48 h. The non-invading cells were removed from the inside of insert with a cotton swab. Cells that invaded to the lower side of the membrane were fixed and stained with Diff-Quik® (Dade Behring, Newark, DE). Inserts were visualized under a light microscope. The experiment was performed three times independently and the representative data of one experiment are shown.

### Western blot analysis

Cells at the density of 2.5 × 10^5^ cells were seeded into each well of 6-well plates and cultured to confluence. They were washed twice with ice-cold PBS and incubated in lysis buffer containing 20 mM Tris-HCl (pH 7.5), 1% Triton X-100, 150 mM NaCl, 10% glycerol, 1 mM Na_3_VO_4_, 50 mM NaF, 100 mM phenylmethylsulfonyl fluoride, and a commercial protease inhibitor mixture (Roche Molecular Biochemicals, Indianapolis, IN) at 4°C for 20 min. Cell lysates were collected and analyzed for protein content using the BCA protein assay kit (Pierce Biotechnology, Rockford, IL). Samples containing 50 μg of cell lysate proteins per lane were resolved under denaturing conditions by 10% sodium dodecyl sulfate-polyacrylamide gel electrophoresis (SDS-PAGE) along with EZ-run pre-stained protein ladder (Fisher Scientific, Pittsburgh, PA) and transferred onto PVDF membranes (Invitrogen, Carlsbad, CA). The transferred membranes were blocked for 1 h in 5% nonfat dry milk in TBST (25 mM Tris-HCl, pH 7.4, 125 mM NaCl, 0.05% Tween 20) and incubated with the appropriate primary antibodies (Cell Signaling Technology, Danvers, MA or Santa Cruz Biotechnology, Dallas, TX) at 4°C overnight. Membranes were washed twice with TBST for 10 min and incubated with horseradish peroxidase-coupled isotype-specific secondary antibodies (Cell Signaling Technology, Danvers, MA) for 1.5 h at room temperature. The immune complexes were detected by enhanced chemiluminescence detection system (Amersham Biosciences, Piscataway, NJ) and quantified using analyst/PC densitometry software (Bio-Rad Laboratories, Hercules, CA).

### H-Ras siRNA transfection

Cells were transfected with pre-designed human H-Ras siRNA (Santa Cruz Biotechnology, Dallas, TX: sc-29340) or control siRNA (Santa Cruz Biotechnology, Dallas, TX: sc-37007), according to the manufacturer's protocol. H-Ras expression in the H-Ras and control siRNA-transfected cells was determined by Western blotting as described above.

### Inhibition of ERK MAPK signaling pathway

SWCNT-exposed cells at 2 × 10^5^ cells were seeded into each well of 6-well plates and cultured for 48 h. Cells were then pre-incubated with the ERK inhibitor U0126 (Cell Signaling Technology, Danvers, MA) at different concentrations for 2 h prior to subjecting to cell invasion or Western blot assays as described above.

### Ingenuity pathway analysis

H-Ras-ERK invasion signaling network was generated using Ingenuity Pathway Analysis (IPA, version Fall 2012; Redwood City, CA) from whole genome expression data of SWCNT-treated mesothelial cells previously deposited on NCBI's Gene Expression Omnibus (GenBank ID: GSE48855) (Lohcharoenkal et al., 2013). Gene signaling networks (GSN) associated with cell invasion were created and mapped. Only genes that have the first order relationship with ERK were kept in the network. Genes were included in the GSN if they promoted invasion and were overexpressed or if they inhibited invasion and were underexpressed.

### Quantitative real-time PCR of epithelial-mesenchymal transition (EMT) transcriptional regulators

The expression of EMT transcriptional regulators including SnaI, Twist, E-cad, N-cad, FN1, and VIM was analyzed in control and SWCNT (0.2 μg/cm^2^)-exposed MeT-5A cells. Briefly, total RNA was isolated from cells using RNeasy mini kit (Qiagen, Valencia, CA), according to the manufacturer's instructions. The extracted RNA was then reverse transcribed into cDNA by high capacity RNA to cDNA kit (Applied Biosystems, Carlsbad, CA). After the reverse transcription reaction was finished, 10 μL of diluted cDNA product (final cDNA quantity 100 ng) was mixed with 10 μL of Taqman® master mix (Applied Biosystems) and transferred into Taqman® array plate (Applied Biosystems). Quantification of the PCR products was performed by NFQ-FAM® method using the Applied Biosystems 7500 Real-Time PCR system with the following profile: 1 cycle at 94°C for 2 min, 40 cycles at 94°C for 15 s, 60°C for 1 min, 72°C for 1 min. Data analysis was performed using the ABI sequence detection software (Applied Biosystems) by relative quantification. The threshold cycle (Ct), which is defined as the cycle at which PCR amplification reaches a significant value, is given as the mean value. The relative expression of each mRNA was calculated by the ΔCt method, where ΔCt is the value obtained by subtracting the Ct value of the housekeeping gene *18S* mRNA from the Ct value of the target mRNA. The amount of the target relative to *18S* mRNA was expressed as 2^−Δ*Ct*^.

### Immunofluorescence staining

Cellular cortactin expression was visualized by immunofluorescence microscopy (Zeiss LSM 510 Axiovert 100 M, Zeiss, Thornwood, NY). Briefly, cells were cultured to confluence on glass cover slips and fixed in 4% paraformaldehyde in PBS. The samples were rinsed three times, permeabilized with 1.2% Triton X-100 for 5 min, rinsed three times and blocked with 1% bovine serum albumin (BSA) in PBS for 1 h before staining with 1:100 cortactin primary antibody (Cell Signaling Technology, Danvers, MA) followed by Alexa Fluor-conjugated secondary antibody (Invitrogen, Carlsbad, CA). The stained cells were mounted with ProLong® gold antifade reagent with DAPI (Invitrogen, Carlsbad, CA) and visualized by fluorescence microscopy. All microscopic exposure conditions were set the same between samples for fluorescence intensity comparison.

### Statistical analysis

All experiments were performed in triplicate. Difference between groups was assessed by One-Way analysis of variance (ANOVA). Differences were considered significant if *P*-values were < 0.05.

## Results

### SWCNT exposure induces aggressive neoplastic-like phenotype of mesothelial cells

**Figure 1:**
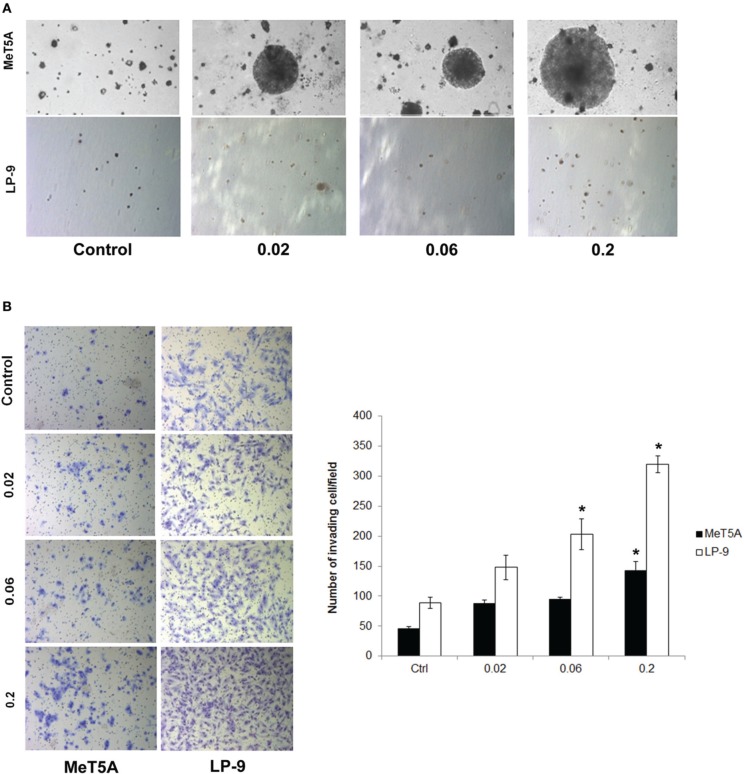
**Cancer hallmark transformation phenotypes of SWCNT-exposed MeT-5A and LP-9 cells**. Anchorage-independent cell growth was determined by soft agar colony formation assay. Two-month exposed MeT-5A and LP-9 cells in culture medium containing 0.33% agar were plated onto the dish coated with 0.5% agar in culture medium. Colonies were examined by light microscopy after 4 weeks of incubation **(A)**. Invasion was assessed in the SWCNT-exposed cells using BD Matrigel® invasion chamber. Invading cells were fixed, stained and visualized under a microscope. The number of invading cells was counted and presented as a bar chart **(B)**. ^*^Significantly difference from control with *P* < 0.05 (*n* = 3).

Cancer hallmark phenotypes were determined in SWCNT-exposed and control MeT-5A and LP-9 cells by established methods. Anchorage-independent cell growth was determined by assessing the size and number of isolated colonies on soft agar. Increased colony size was observed in the MeT-5A cells exposed to all doses of SWCNT with the highest dose (0.2 μg/cm^2^) inducing the biggest colonies (Figure 1A). At the same dose range, SWCNT had minimal effect on colony formation in LP-9 cells (Figure 1A), possibly due to their decreased susceptibility and limited lifespan under non-adherent conditions.

The invasiveness of SWCNT-exposed mesothelial cells was assessed by Transwell® invasion assay. SWCNT was able to increase the invasiveness of both MeT-5A and LP-9 cells in a dose-dependent manner as compared to their passage-matched control cells (Figure 1B). At the highest exposure dose (0.2 μg/cm^2^), significant increase in the number of invading cells was observed in both cell types. These results indicate the invasion-promoting activity of SWCNT in mesothelial cells.

### H-Ras overexpression in SWCNT-exposed mesothelial cells

**Figure 2:**
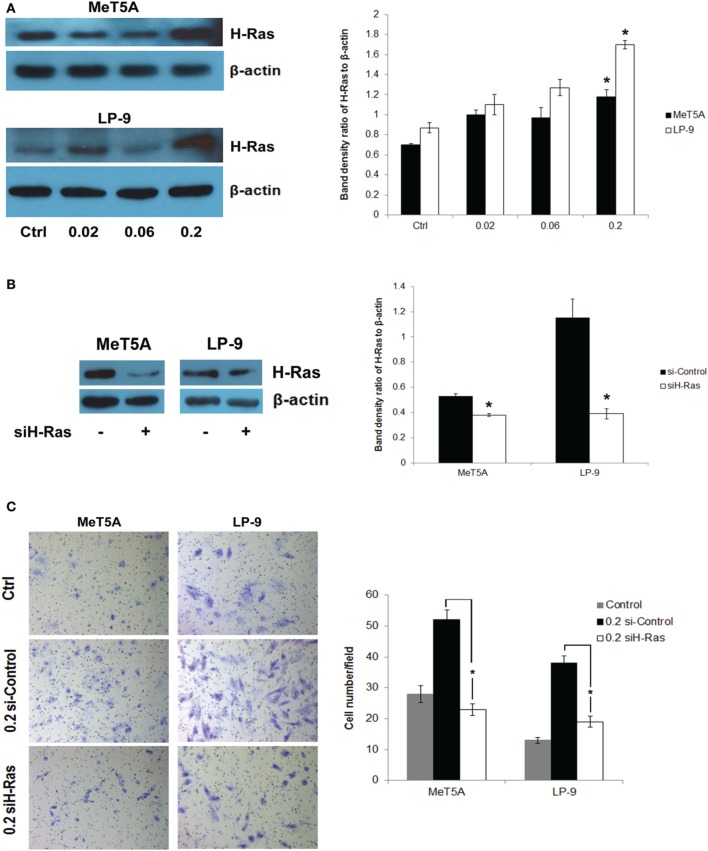
**Up-regulation of H-Ras in SWCNT-exposed mesothelial cells**. Total H-Ras protein expression in SWCNT-exposed MeT-5A and LP-9 cells were analyzed by Western blotting. Band densities were determined and presented as a bar chart. Overexpression of H-Ras was observed in both SWCNT-exposed cell types at the high dose of 0.2 μg/cm^2^ **(A)**. SWCNT (0.2 μg/cm^2^)-exposed MeT-5A and LP-9 cells were transfected with siH-Ras or siControl RNA, and H-Ras protein expression was determined by Western blotting **(B)**. Cell invasion assays showed a reduced invasive capacity of the siH-Ras cells as compared to siControl cells **(C)**, ^*^significantly difference with *P* < 0.05 (*n* = 3).

We hypothesized that H-Ras expression and its downstream targets may be activated and play a role in the neoplastic transformation of SWCNT-exposed mesothelial cells. To test this possibility, we analyzed H-Ras (also known as transforming protein p21) expression in SWCNT-exposed and control cells by Western blotting. Increased expression of H-Ras was observed in the SWCNT-exposed MeT-5A and LP-9 cells as compared to control cells (Figure 2A). The importance of H-Ras overexpression on the aggressive behavior of SWCNT-exposed cells was evaluated by siRNA silencing experiments. SWCNT-exposed MeT-5A and LP-9 cells were transfected with siRNA against H-Ras (siH-Ras) or control siRNA (siControl), and subjected to cell invasion assays. Figures 2B,C show that the siH-Ras treatment resulted in a substantial reduction of the H-Ras expression and a parallel decrease in cell invasion activity as compared to the siControl treatment.

### Activation of downstream signaling pathway of H-Ras

**Figure 3:**
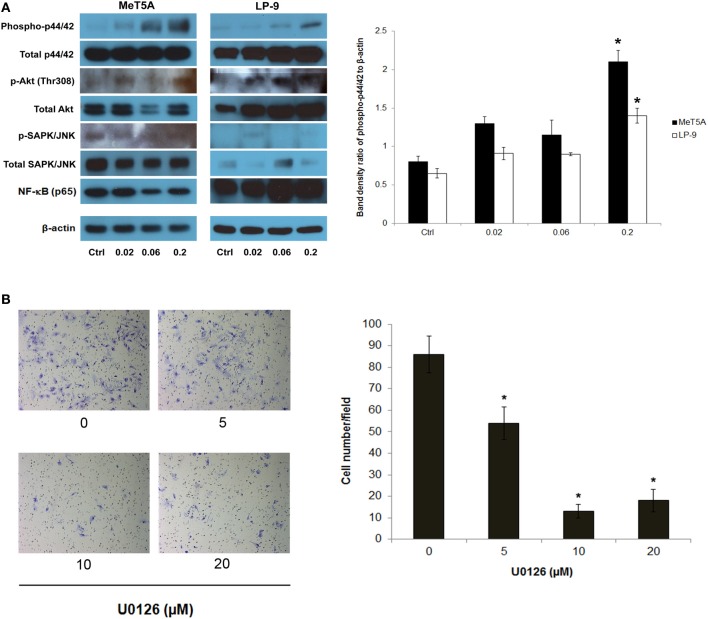
**Analysis of downstream effectors of H-Ras in SWCNT-exposed MeT-5A and LP-9 cells**. A dose-dependent activation of p44/42 (ERK1/2) was evident in the SWCNT-exposed cells **(A)**. U0126, an ERK kinase inhibitor, was used to confirm the importance of ERK activation in the invasiveness of SWCNT-exposed cells. SWCNT (0.2 μg/cm^2^)-exposed Met-5A cells were treated with various concentrations (5–20 μM) of U0126 for 2 h, after which they were washed and subjected to Transwell® invasion assay. A substantial and dose-dependent decrease in the number of invading cells was evident in the treatment groups **(B)**, ^*^significantly difference from no treatment control with *P* < 0.05 (*n* = 3).

To dissect the signaling pathway downstream of H-Ras that may be involved in the neoplastic phenotype of SWCNT-exposed cells, the activation of several known effectors of *Ras* including ERK, JNK, Akt, and NF-κB was investigated. Among these, p44/42 (ERK1/2) was shown to be prominently activated in the SWCNT-exposed cells, suggesting the possible role of ERK signaling in the aggressive phenotype of these cells (Figure 3A).

To assess the functional importance of activated p44/42 in the aggressive phenotype, SWCNT-exposed cells were treated with non-cytotoxic doses of U0126, a selective ERK kinase inhibitor, and analyzed for cell invasiveness by Transwell® invasion assay. The results showed that the ERK inhibitor effectively inhibited the invasive activity of the cells in a dose-dependent manner (Figure 3B). These results support the role of ERK signaling downstream of H-Ras activation in SWCNT-induced cell invasiveness.

### Potential genes associated with ERK signaling in SWCNT-exposed mesothelial cells

**Figure 4:**
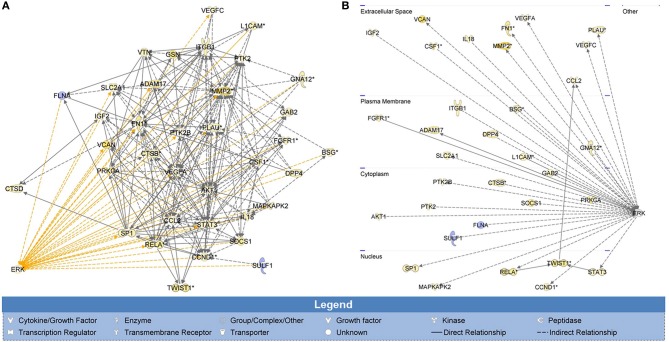
**Whole genome analysis of cell invasion network in SWCNT-exposed mesothelial cells**. H-Ras-ERK invasion signaling network was created from whole genome expression data previously deposited on NCBI's Gene Expression Omnibus (GenBank ID: GSE48855) using IPA. Gene signaling networks (GSN) associated with cell invasion were created and mapped. Genes were included in the GSN if they promoted invasion and were overexpressed or if they inhibited invasion and were underexpressed. Invasion GSN with first order relationship to ERK **(A)** and a cross-section diagram for location of each gene within the cells **(B)** are shown. *MMP2, PLAU, AKT, CCND1, ITG*, several inflammatory genes and EMT-related genes (*TWIST, FN1*) were found to be the up-regulated genes with first relationship with ERK in the GSN. Most of these genes were reported to involve in cell motility and metastasis process.

To determine the possible effectors of H-Ras-ERK signaling in the tested cells, whole genome expression data of SWCNT-exposed mesothelial cells from our previous study was subjected to IPA. Invasion GSN with first order relationship to ERK and a cross-section diagram for location of each gene within the cells are shown in Figure 4. Genes of potential interest to the SWCNT-induced cell invasiveness include some proteolytic enzyme-encoding genes (*MMP2, PLAU*), *AKT*, cyclin D1, integrin, several inflammatory genes, and EMT related genes (*TWIST, FN1*).

### Integrin alpha v and cortactin overexpression in SWCNT-exposed mesothelial cells

**Figure 5:**
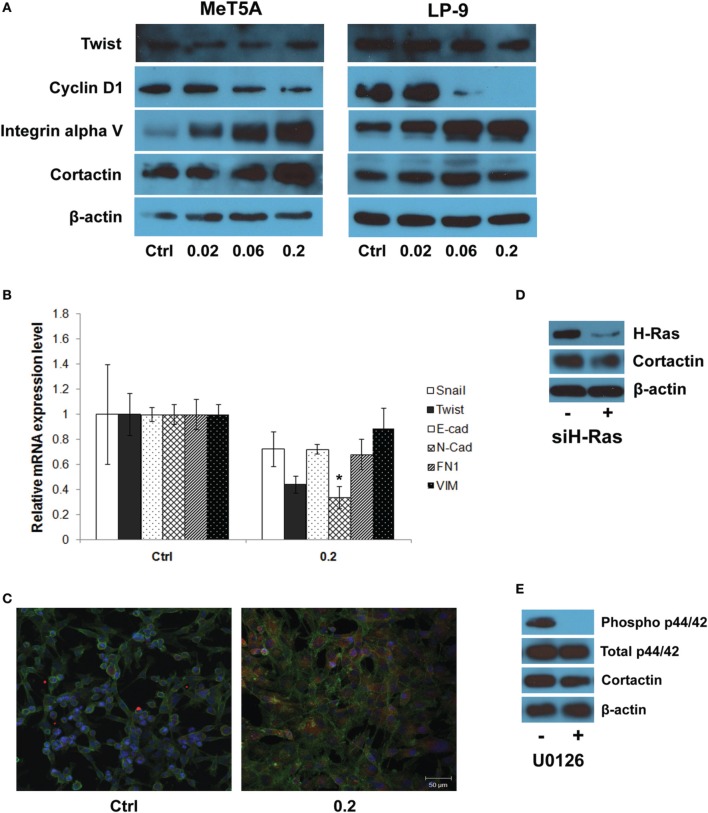
**Molecular and functional assays of genes in the gene signaling network**. Western blot analysis showed a dose-dependent increase in integrin alpha V and cortactin expression in SWCNT-exposed MeT-5A and LP-9 cells **(A)**. Real-time PCR analysis showed a down-regulation of all EMT transcriptional regulators in SWCNT (0.2 μg/cm^2^)-exposed MeT-5A cells compared to control cells **(B)**. Immunofluorescence staining showed a substantial increase in cortactin protein expression in SWCNT (0.2 μg/cm^2^)-exposed MeT-5A cells compared to control cells **(C)**. Down-regulation of H-Ras by siH-Ras **(D)** and inactivation of ERK by U0126 **(E)** lowered the expression of cortactin in SWCNT-exposed MeT-5A cells. ^*^Significantly difference from no treatment control with *P* < 0.05 (*n* = 3).

Further investigations on the genes from the invasion GSN were performed using Western blotting and quantitative real-time PCR. Western blot analysis showed a dose-dependent increase in integrin alpha V expression in SWCNT-exposed MeT-5A and LP-9 cells (Figure 5A). Cortactin, a protein known to be involved in cell motility and important in neoplasia development, was also overexpressed in the SWCNT-exposed cells. PCR analysis of EMT transcriptional regulators showed a repression of all EMT regulators examined in the exposed cells (Figure 5B). The relationship between cortactin and H-Ras-ERK activation was further evaluated by immunofluorescence staining. The results showed that cortactin was substantially induced in the SWCNT-exposed cells, consistent with the H-Ras/ERK data (Figure 5C). Overexpression of H-Ras in the MeT-5A cells also resulted in the up-regulation of cortactin (Supplementary Figure 1A), whereas siRNA downregulation of H-Ras decreased the cortactin expression in SWCNT-exposed cells (Figure 5D). In addition, the ERK kinase inhibitor U0126 mitigated the cortactin expression as analyzed by Western blotting (Figure 5E).

## Discussion

Rapid increase in CNT production and utility has raised a concern over the potential adverse effects of CNTs on human health and the environment. Both *in vivo* and *in vitro* studies have provided useful information on the biological and toxicological effects of CNTs; however, most of these studies have focused on short-term acute effects of the nanomaterials, which is due in part to the lack of appropriate experimental models for long-term studies. We have developed a chronic cellular exposure model to study the long-term biological effects of nanomaterials in various lung cell types including human bronchial epithelial cells (BEAS-2B), human small airway epithelial cells (SAEC), and human pleural mesothelial cells (MeT-5A) (Wang et al., 2011, 2014; Lohcharoenkal et al., 2013). Neoplastic transformation of these cells was demonstrated upon chronic exposure to low-dose CNTs as demonstrated by their anchorage-independent cell growth, apoptosis resistance, increased cell motility and angiogenesis. The induction of proto-oncogenes and cell division anomalies including centrosome fragmentation, mitotic spindle disruption, and aneuploidy has also been reported in CNT-exposed cells (Sargent et al., 2009; Wang et al., 2014). Despite its established importance in lung carcinogenesis, the role of H-Ras in CNT-induced carcinogenesis has not been reported, although a recent study indicated the involvement of H-Ras signaling in DNA damage caused by CNTs (Tong et al., 2011). Thus, the role of H-Ras in the neoplastic transformation of human mesothelial cells was focused in this study.

Two types of human mesothelial cells (MeT-5A and LP-9) were continuously exposed to different concentrations (0.02, 0.06, and 0.2 μg/cm^2^) of SWCNT for 2 months and analyzed for cancer hallmark phenotypes. As shown in Figure 1, SWCNT induced colony formation and cell invasion in MeT-5A cells, whereas it induced mainly cell invasion with minimal effect on colony formation in LP-9 cells. Anchorage-independent cell growth is the most commonly used *in vitro* indicator of malignant transformation and it correlates well with *in vivo* tumorigenicity (Risser and Pollack, 1974; Shin et al., 1975). This indicates malignant transformation of SWCNT-exposed MeT-5A cells, although the control MeT-5A cells also formed slow-growing colonies on soft agar. This could be due to the fact that the cells were immortalized by transfection with the pRSV-T plasmid (an SV40 ori- construct containing the SV40 early region and sarcoma virus long terminal repeat). ATCC indicates that this cell line can form colonies in a semi-solid medium but is non-tumorigenic in immunocompromised mice. Therefore, the colony formation observed in the control cells is due to indigenous properties of the cells, and not because of their tumorigenicity. For normal cells, spontaneous colony formation was concentration dependent and the threshold for colony formation varied between cell types and some exogenous source of colony stimulating factor (CSF) may be needed. In case of normal mesothelial cells such as LP-9 cells, high concentrations of epidermal growth factor (EGF) and hydrocortisone in addition to fetal calf serum were required to induce colony formation in semi-solid medium (La Rocca and Rheinwald, 1985). Cell invasion is a crucial step in many physiological processes and its impairment has been implicated in many pathological disorders such as tumor growth and metastasis. It has been used to assess the aggressive and malignant phenotypes of cells (Cho and Klemke, 2000). Our results demonstrated the induction of aggressive cancer phenotypes by SWCNT in mesothelial cells.

H-Ras expression was examined in SWCNT-exposed cells and was found to be elevated in both the SWCNT-exposed MeT-5A and LP-9 cells (Figure 2A). The functional importance of H-Ras overexpression was demonstrated by colony formation assay in H-Ras-transfected MeT-5A cells (Supplementary Figure 1B). Since overexpression of H-Ras has been shown to be important in cancer cell invasion and progression (Theodorescu et al., 1990), we hypothesized that H-Ras overexpression and the activation of its downstream targets may be crucial to the invasive transformation of SWCNT-exposed mesothelial cells. The results of our H-Ras knockdown experiments support this notion (Figures 2B,C).

Downstream effectors of *Ras* include members of the MAPK family comprising of JNK, ERK and stress-activated protein kinase-2 (p38). MAPKs are among the major kinases that transduce extracellular signaling into cellular responses and play a pivotal role in the regulation of cell proliferation, apoptosis, differentiation, cytoskeleton remodeling, and cell cycle regulation (Brunet and Pouyssegur, 1996; Foltz et al., 1997). Key effectors of Ras including ERK, JNK, AKT, and NF-κB were examined in SWCNT-exposed MeT-5A and LP-9 cells. An activation of ERK1/2 (p44/42) was prominent in these cells (Figure 3A), suggesting the possible role of H-Ras-ERK signaling in the invasiveness of SWCNT-exposed mesothelial cells. Our results on the inhibitory effect of U0126 ERK inhibitor on the invasivity of SWCNT-exposed MeT-5A cells support this notion (Figure 3B). Consistent with this finding, several studies have indicated the involvement of ERK in the migration and invasion of various forms of cancer including glioblastoma, pancreatic carcinoma and lung cancer (Lakka et al., 2000; Lu et al., 2011).

Invasion GSN obtained from the whole genome expression data of SWCNT-exposed mesothelial cells was created and filtered for ERK-related genes as shown in Figure 4. *MMP2, PLAU, AKT, CCND1, ITG*, several inflammatory genes and EMT-related genes (*TWIST, FN1*) were all up-regulated and exhibited the first relationship with ERK. *MMPs* are signature invasion marker genes that encode proteins involved in the degradation of ECM and are typically highly active during cancer development and progression (Passlick et al., 2000). MMPs belong to a family of zinc-dependent endopeptidases which are divided into different classes. MMP-2 (gelatinase A) has an important role in basement membrane turnover due to its specific activity to collagen type IV or gelatin. Its degradation plays a role in cell invasion of the vasculature and is considered to have a key role in metastasis. *MMP-2* expression has been associated with the invasiveness of many cancer cell lines and is elevated in high-grade tumors, specifically at the invasive front and in vascular invasion (Birkedal-Hansen et al., 1993; Coussens and Werb, 1996). Our previous study has demonstrated the importance of MMP-2 in cell invasion induced by chronic CNT and asbestos exposure (Lohcharoenkal et al., 2013). Urokinase-plasminogen activator or *PLAU* gene is known to encode uPA, the protease which degrades ECM and plays critical roles in cell migration, tissue remodeling, angiogenesis, tumor invasion, and metastasis (Suzuki et al., 2004). Plasminogen activators convert plasminogen to plasmin, which works efficiently in proteolysis of the fibrin. In addition, plasminogen activators activate several MMPs and could also facilitate the MMP activities. A positive feedback loop between the binding of uPA to uPA receptor (uPAR) and Ras-ERK signaling pathway activation has been reported in many cell types and implicated in cell migration and progression of cancer (Ma et al., 2001). AKT (also known as protein kinase B or PKB) is a serine/threonine kinase that is involved in mediating various biological responses, such as inhibition of apoptosis and stimulation of cell proliferation. There are three highly related isoforms of AKT (AKT1, AKT2, and AKT3) and these represent the major signaling arm of PI3K (Park et al., 2001). MEK/ERK and PI3K/AKT pathways are often concurrently activated by separate genetic alterations in cancer cells but it was reported that ERK and AKT signaling cooperate to translationally regulate metastatic progression of certain types of cancer, e.g., colorectal cancer (Ye et al., 2013). Integrins are cell surface receptors that interact with ECM and mediate intracellular signals that regulate many cellular processes including cell shape, mobility and progression through the cell cycle (Hynes, 2001). Integrins play an important role in cell signaling by affecting the cell signaling pathways of protein kinases including Ras-Raf-MEK-ERK pathway (Schlaepfer et al., 1994). Numerous studies have shown that integrin expression profiles are subject to change during cancer growth and progression and that such change contributes to the aggressive behavior of cancer cells (Danen and Sonnenberg, 2003; Danen, 2005). The enhanced expression of integrin is reported to be associated with EMT and poor prognosis of cancer (Bates et al., 2005).

Western blot and real-time PCR studies demonstrated the up-regulation of integrin alpha V, but not EMT transcriptional regulators, in SWCNT-exposed mesothelial cells (Figures 5A,B). Since integrin/focal adhesion kinase (FAK)/cortactin has been reported to regulate cell motility and proliferation, cortactin expression in the SWCNT-exposed cells was examined and found to be upregulated in the SWCNT-exposed MeT-5A and LP-9 cells (Figure 5A). Cortactin is a multidomain adapter protein, essentially contributing to cortical actin regulation. Regulation of this pool of actin is controlled by a variety of actin regulatory proteins at integrin or cadherin adhesion sites and is important in many normal and pathological cellular processes, such as adhesion, migration, morphogenesis, tumor progression and metastasis (Weed and Parsons, 2001; Clark et al., 2007). Cortactin functions in actin assembly via interaction with actin-related protein-2/3 (Arp2/3) complex, which is dependent on Src-mediated phosphorylation of cortactin (Schubert and Dotti, 2007).

Besides tyrosine phosphorylation, cortactin is a target for multiple serine/threonine kinases (Martin et al., 2006). Stimulation of tumor cells with EGF leads to phosphorylation of serine residues 405 and 418, coincident with a characteristic shift in cortactin electrophoretic mobility from 80 to 85 kDa in SDS-PAGE. The mobility shift and phosphorylation of S405/S418 are impaired by pharmacologic inhibition of mitogen activated protein/extracellular signal regulated kinase (MEK)1/2. Biochemical evidence indicates that the MEK effector kinases ERK1/2 directly phosphorylate cortactin at these sites (Campbell et al., 1999). Thus, the relationship between cortactin and H-Ras-ERK activation in SWCNT-exposed cells was evaluated and the positive correlation between H-Ras and cortactin expression was observed (Figure 5C and Supplementary Figure 1A). In good agreement, knockdown of H-Ras or chemical inhibition of ERK kinase decreased the level of cortactin expression in the SWCNT-exposed cells (Figures 5D,E).

In summary, we demonstrated that prolonged exposure of human mesothelial cells to SWCNT induced aggressive neoplastic-like transformation in concomitant with H-Ras up-regulation. knockdown and overexpression studies indicated the relationship between H-Ras expression and the invasive phenotype of SWCNT-exposed cells. ERK1/2 was identified as an important effector for the aggressive phenotype of the cells as indicated by their reduced invasiveness by ERK kinase inhibitor. Whole genome microarray and Western blot analyses indicated the possible involvement of integrin alpha V in the H-Ras-ERK invasion signaling. Additionally, cortactin was shown to be a downstream target of H-Ras-ERK signaling in the SWCNT-exposed cells.

## Author contributions

Warangkana Lohcharoenkal designed and performed cellular and molecular studies, and prepared the manuscript. Liying Wang prepared nanoparticle preparations and performed chronic exposure. Todd A. Stueckle performed microarray and Ingenuity Pathway Analysis. Jino Park performed real-time PCR experiments. William Tse participated in the design of the study and provided research reagents. Cerasela-Zoica Dinu characterized nanomaterials. Yon Rojanasakul designed and coordinated the project, and prepared the manuscript. All authors read and approved the final manuscript.

## Disclaimer

The findings and conclusions in this report are those of the authors and do not necessarily represent the views of the National Institute for Occupational Safety and Health.

### Conflict of interest statement

The authors declare that the research was conducted in the absence of any commercial or financial relationships that could be construed as a potential conflict of interest.

## Abbreviations

SWCNT: single-walled carbon nanotubes
ERK: extracellular signal-regulated kinases
MAPK: mitogen-activated protein kinases
PI3K: phosphatidylinositol 3-kinase
MMP: matrix metalloproteinase
EGF: epidermal growth factor
GSN: gene signaling network
IPA: ingenuity pathway analysis
E-cad: E-cadherin
N-cad: N-cadherin
FN: fibronectin
VIM: vimentin
FAK: focal adhesion kinase
uPA: urokinase-plasminogen activator
BEAS-2B: human bronchial epithelial cells
SAEC: human small airway epithelial cells
MeT-5A: immortalized human pleural mesothelial cells
LP-9: normal human peritoneal mesothelial cells.
