# Supplementary material for: Role of H-Ras/ERK signaling in carbon nanotube-induced neoplastic-like transformation of human mesothelial cells
Source: Front Physiol. 2014 Jun 12;5:222. doi: 10.3389/fphys.2014.00222 (PMC4054652; doi:10.3389/fphys.2014.00222)
Supplement: Supplementary file 1 [file Presentation1.ZIP › Supp Fig1.DOC]

**Supplementary figure 1**

Mesothelial MeT-5A cells were transfected with control or H-Ras plasmid, and analyzed for H-Ras and coratactin protein expression by Western blotting. Positive correlation between H-Ras and cortactin expression was observed (**A**). H-Ras-overexpressing MeT-5A cells were analyzed for anchorage-independent growth by soft agar colony formation assay. Increased colony size was observed in H-Ras-overexpressing MeT-5A cells (**B**).
